# An Uncommon Presentation of Mucopolysaccharidosis Type IIIb

**Published:** 2019

**Authors:** Alireza REZAYI, Mohammad FESHANGCHI-BONAB, Reza TAHERIAN

**Affiliations:** 1 Department of Pediatric, Loghman Hospital, Shahid Beheshti University of Medical Sciences, Tehran, Iran; 2 Department of Pediatric, Mofid Children's Hospital, Shahid Beheshti University of Medical Sciences, Tehran, Iran; 3 School of Medicine, Shahid Beheshti University of Medical Sciences, Tehran, Iran

**Keywords:** Mucopolysaccharidosis type III, Landau-Kleffner syndrome, Electroencephalography

## Abstract

Mucopolysaccharidosis type III (MPS III; Sanfilippo syndrome) is a metabolic disorder characterized by a lysosomal enzyme deficiency in the catabolic pathway of heparan sulfate. The patients with mucopolysaccharidosis type III usually present with declined neurocognitive functions such as speech and hearing loss. Subtle somatic features of patients with mucopolysaccharidosis type III can lead to diagnostic delay and consequently, a greater neurocognitive deterioration may happen. Herein, we report a 9-yr-old boy referred to Loghman Hospital, Tehran, Iran, in 2018. He had developed normally up to four yr of age when his symptoms initiated with behavioral disturbances such as auditory agnosia and decreased verbal communication. Progression of his symptoms to seizure and ataxia, brain perfusion scan and electroencephalography features strongly suggested landau-Kleffner syndrome. However, results of gene sequencing analysis and high urinary glycosaminoglycan excretion confirmed mucopolysaccharidosis type III as his final diagnosis. This case strongly recommends screening for metabolic disorders such as mucopolysaccharidosis type III in the patients diagnosed as having landau-Kleffner syndrome.

## Introduction

Mucopolysaccharidosis (MPS) type III, also known as Sanfilippo syndrome, is an autosomal recessive neurodegenerative condition associated with a deficiency in lysosomal enzyme catalyzing the catabolic pathway of glycosaminoglycan (GAG) heparan sulfate. Based on the different enzyme deficiency in the mentioned pathway, MPS III has four subtypes named from A to D (1, 2). 

The total incidence of MPS III varies between 0.28 and 4.1 per 100 000 live births with types A and B, being more prevalent than types C or D (1). MPS type IIIB is specifically caused by deficiency of α-N-acetylglucosaminidase resulted by mutation in NAGLU (3). 

MPS IIIB presents with central nervous system involvement characterized by progressive speech delay, hearing loss, behavioral problems such as hyperactivity and sleep disturbances with subtle somatic features in a child with an initial period of normal development (2). These features may also be closely related to attention-deficit/hyperactivity disorder (ADHD). Hence, children with MPS IIIB may be misdiagnosed as having ADHD; however, they do not respond to stimulant medication (1, 2). The Landau-Kleffner syndrome (LKS) is also associated with attentional problems and hyperactivity as the most common behavioral disturbances of this syndrome (4). LKS usually presents at the age of 3-10 yr (most commonly in 5-7 yr) in a child with previous normal development with neurological symptoms including acquired auditory agnosia, regression of speech and epileptiform pattern in EEG (5). 

Here, we report a rare case of a boy presented with typical features of landau Kleffner syndrome but the final diagnosis was unexpectedly MPS IIIB. To our knowledge, LKS is not reported to present similarly to MPS in any previous reports. Since a delayed diagnosis of MPS can result in a progressed neurocognitive decline, this case highlights the importance of considering metabolic disorders, such as MPS III, in children with speech loss even in the presence of epilepsy. 

## Case Report

A 9-yr-old boy was referred to Loghman Hospital, Tehran, Iran, in 2018, with speech regression, seizure, and ataxia. He was developmentally normal until the age of 4 yr when his symptoms initiated with behavioral disturbances including attention deficit, auditory agnosia, decreased verbal communication, speech repetition, and IQ deterioration. He had first episodes of generalized tonic-clonic seizure with upward gaze and jaw locking during sleep and also intermittent urinary incontinence when he was 6 yr old. The child started symptoms of autism spectrum disorder such as echolalia, poor eye to eye contact and repetitive behavior.

**Figure 1:**
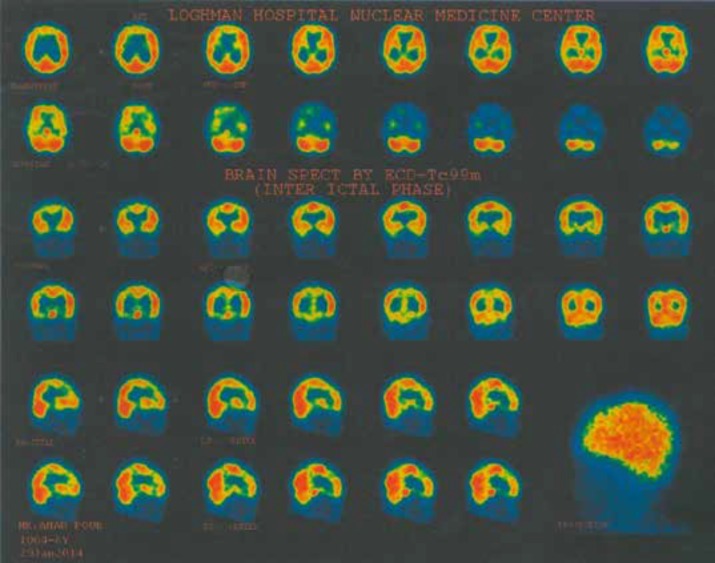
Brain perfusion SPECT with decreased perfusion in left centrotemporal lobe

**Figure 2:**
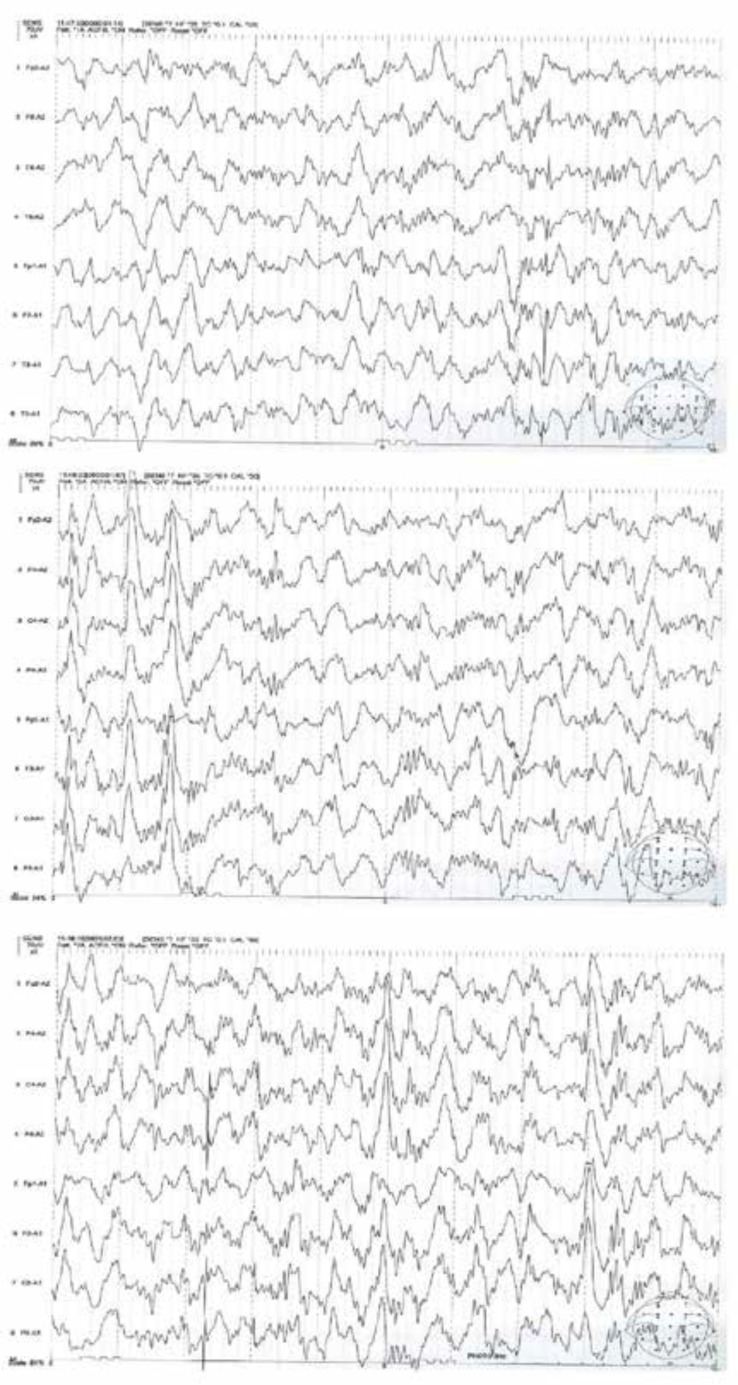
Electroencephalography showing bilateral synchronous spike-wave complex in the temporal regions

On neurological examination, the patient had no focal signs. Motor and sensory examination of four extremities were intact. Primary laboratory studies included blood cell count (CBC), renal and liver function tests were normal. CSF examination was within normal limit. The child had normal brain MRI and auditory brainstem response (ABR). Brain perfusion SPECT showed decreased perfusion in left centrotemporal lobe (Figure 1). Amplitude EEG showed bilateral synchronous spike-wave complex in the temporal regions (Figure 2).

**Figure 3:**
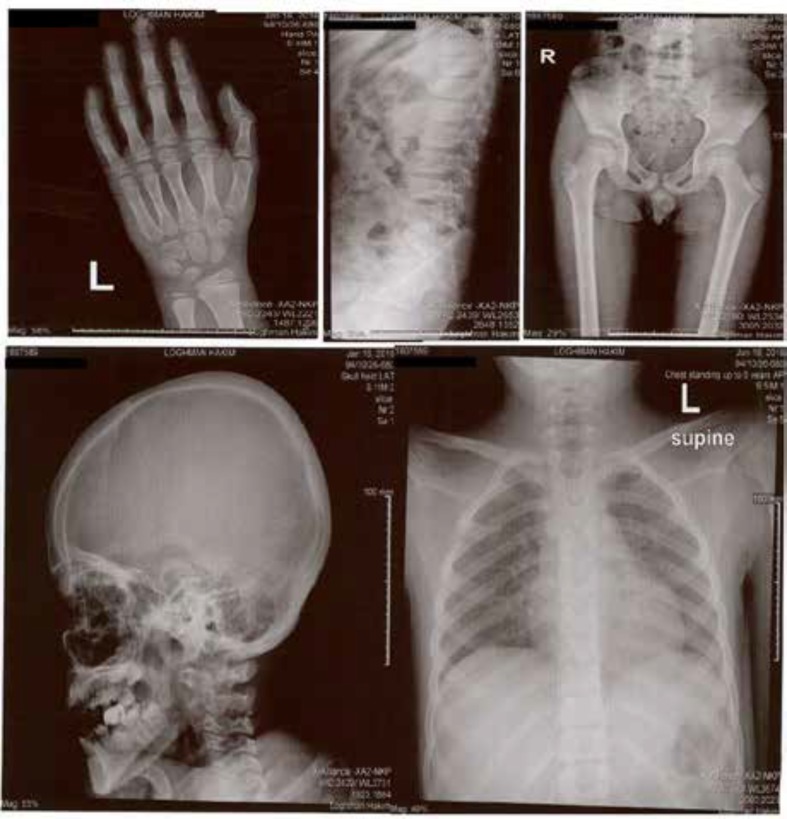
Radiographic evaluation of hand and wrist, lumbar spine, pelvis, head and neck and thorax

On the basis of presentation, EEG and SPECT study, LKS diagnosis was confirmed and methylprednisolone, valproate sodium, levetiracetam, IVIG and speech therapy were initiated; however, the patient continued generalized tonic-clonic seizure in sleep, severe behavioral disorders and aphasia. Due to poor response to treatment, symptoms progression and relative parents, whole exome sequencing was performed, which revealed mutation in exon 5 of NAGLU gene suggesting MPS IIIB. Although the patient had not any sign of coarse face or dysostosis multiplex (Figure 3), high urinary glycosaminoglycan excretion confirmed MPS III diagnosis. 

Informed consent was taken from patients parents. 

The study was approved by the Ethics Committee of Shahid Beheshti University of Medical Sciences, Tehran, Iran. 

## Discussion

There is a large variability in the clinical spectrum of MPS III which may be a result of residual enzyme activity caused by different gene mutations (6). While the patients with MPS III have an apparently normal development in first years of their life, they eventually decline in neurocognitive abilities such as speech and hearing. The presence of somatic symptoms, such as hepatomegaly, in other types of MPS, help the diagnosis of this disorder; however, there are subtle somatic symptoms in patients with MPS III (7). This results in diagnostic delay in MPS III population leading to a greater neurocognitive deterioration in these patients which may result in seizure in the final stages (2). Therefore, while MPS III may be misdiagnosed as an autism spectrum disorder in its early phase, the emergence of the seizure may lead MPS III to be misdiagnosed as epileptic syndromes. 

Landau-Kleffner syndrome is an epileptic syndrome classified among the epileptic encephalopathies, i.e. conditions in which epileptiform abnormalities result in progressive cognitive dysfunction (8). EEG remains the gold standard for the diagnosis of LKS demonstrating focal or multifocal spike-wave complexes predominantly over temporal regions (4). Landau-Kleffner syndrome may represent a final common pathway with multiple etiologies such as metabolic disorders, but many of them are yet unknown (9). Indeed, diagnosis of the LKS in a patient may rise the need of searching for other syndromes which result signs and symptoms similar to LKS. Our case, for the first time, showed that MPS III might be one of these syndromes.

The MPS III is a rare disorder and its diagnosis is mainly based on the laboratory and genetic findings, hence limited studies have investigated the EEG features of patients with MPS III. Diffuse slowing without epileptiform abnormalities in the rest was the most common feature in the EEG of the early course of MPS III (10). In Brazil, a clear predominance of nonspecific EEG abnormalities was revealed in patients with MPS; however, epileptogenic paroxysms may be found in children with MPS with or without epilepsy (11). Hence, this study suggests performing EEG routinely during the neurological examination of patients with MPS (11). Our case adds to current understanding of EEG features of MPS III showing that spike-wave complex may be an EEG feature of a progressed MPS III. 

MRI is the primary imaging technique to detect CNS alterations in patients with MPS. Although a large spectrum of findings can be recognized, from negligible to severe, common features of brain MRI in patients with MPS include brain atrophy, the abnormal signal intensity in the white matter and dilatation of periventricular spaces and third ventricle (12). In our case, however, the patient had a normal brain MRI even with progressed neurological disorders. Although the presence of a normal MRI with a progressed MPS disease is a novel finding in our case, it is consistent with previous reports that emphasize no association between MRI findings and clinical status of the disease (12). An extensive review of the literature did not yield any reports of brain perfusion SPECT in patients with MPS. As the first report, a decreased perfusion in left centrotemporal lobe was noted in brain perfusion SPECT in our case suggesting the potential benefit of SPECT study in evaluating patients with MPS III. Neurophysiologic assessment of our patient, including brain MRI, was not consistent with MPS diagnosis, underscoring the laboratory and genetic studies as gold standards for diagnosis of MPS III.

**In conclusion,** given the heterogeneity of LKS, we propose considering other etiologies, such as metabolic disorders, in the patients diagnosed as having LKS. This case showed that MPS III might be one of these metabolic disorders.
